# Stability of the CAG Tract in the *ATXN2* Gene Depends on the Localization of CAA Interruptions

**DOI:** 10.3390/biomedicines12081648

**Published:** 2024-07-24

**Authors:** Oksana Lyasota, Anna Dorohova, Jose Luis Hernandez-Caceres, Alexandr Svidlov, Elena Tekutskaya, Mikhail Drobotenko, Stepan Dzhimak

**Affiliations:** 1 Department of Biologically Active Substances, Kuban State University, 350040 Krasnodar, Russia; 4098789@mail.ru (O.L.); mdrobotenko@mail.ru (M.D.); 2 Laboratory of Problems of Stable Isotope Spreading in Living Systems, Southern Scientific Center of the Russian Academy of Sciences, 344006 Rostov-on-Don, Russia; svidlov@mail.ru (A.S.); tekytska@mail.ru (E.T.); jimack@mail.ru (S.D.); 3 Neurodevelopment Branch, Cuban Center for Neurosciences, Havana 11600, Cuba; kacerjlh@gmail.com; 4 Department of Radiophysics and Nanotechnology, Kuban State University, 350040 Krasnodar, Russia

**Keywords:** *ATXN2* gene, CAG tract, CAA interruptions, parkinsonism, diseases of trinucleotide repeat expansion, DNA model, torque

## Abstract

It is known that the presence of CAA codons in the CAG tract affects the nature and time of disease onset caused by the expansion of trinucleotide repeats. The mechanisms leading to the occurrence of these diseases should be sought not only at the level of the physiological role of the *ATXN2* protein, but also at the DNA level. These mechanisms are associated with non-canonical configurations (hairpins) that can form in the CAG tract. The tendency of hairpins to slide along the corresponding threads is usually considered important to explain the expansion of the CAG tract. At the same time, hairpins occur in areas of open states. Previous studies on the role of CAA interruptions have suggested that, under certain conditions, they can stabilize the dynamics of the hairpin, preventing the expansion of the CAG tract. We calculated the probability of additional open state zones occurrence in the CAG tract using an angular mathematical model of DNA. The calculations made it possible to establish that CAA interruptions affect the stability of the CAG tract, and this influence, depending on the localization of the interruption, can both increase and decrease the stability of the CAG tract.

## 1. Introduction

Dynamic mutations in human genes associated with trinucleotide repeats can cause severe neurodegenerative and neuromuscular disorders known as trinucleotide repeat expansion diseases [1,2,3,4,5]. Among them, a special place is occupied by diseases associated with CAG trinucleotide repeats [6,7].

The trinucleotide repeat region within the first exon of the *ATXN2* gene consists of CAG repeat sequences that form the polyglutamine (polyQ) tract [8]. In healthy individuals, the *ATXN2* polyQ tract typically consists of approximately 22–23 CAG repeats, usually interrupted by one or more CAA trinucleotides [9,10].

Significant expansion of this tract (greater than 34 CAG repeats) is the genetic cause of spinocerebellar ataxia 2 (SCA2), intermediate expansions of the *ATXN2* polyQ tract have been associated with parkinsonism, and some *ATXN2* polyQ expansions have been associated with ALS [7,9,11,12,13,14,15,16,17].

The mechanisms that cause diseases with an increase in the length of the CAG tract have not been fully identified [7,14,18]. A number of authors associate such mechanisms with non-canonical configurations that can be formed in the CAG tract during replication, transcription, or repair [19,20,21]. As is known, during these processes, duplexes unwind and DNA experiences torsional stress [22,23], and as a result, its chains separate. Separated chains containing CAG repeats can form various secondary structures that affect the reading of genetic information [16,17,18,24,25,26].

Interruptions play an important role in pathologies associated with the polyQ tract. Thus, interruption of the pathologically expanded CAG tract by one or more CAA codons can manifest itself as parkinsonism [9,13,27]. To clarify the influence of CAA interruptions on the shape and dynamics of secondary structures in the polyQ tract, experimental and theoretical studies were carried out [9,17,25,27].

In particular, it has been shown that CAA interruptions in the CAG tract can significantly reduce chain slippage in the corresponding hairpins, thereby stabilizing the dynamics of the hairpin [17].

In our work [28], it was established that when torque influences the first exon of the *ATXN2* gene, additional OS zones of significant size can form in the CAG tract, which can provide the formation of secondary structures or otherwise affect the reading of genetic information. Calculations showed that the inverse of the probability of the occurrence of additional OS zones correlates with the age of onset of SCA2 disease.

In this work, using the angular mathematical model of the DNA molecule, we want to show that CAA interruptions affect the stability of the CAG tract of the *ATXN2* gene and that the nature of this influence depends on the location of the interruption.

## 2. Mathematical Model

To study the internal mobility, formation, and dynamics of open state (OS) zones in the DNA molecule, we will use the angular model [29,30], which is based on the analogy in which a double-stranded DNA molecule and a mechanical system consists of two chains of interconnected pendulums and is a system of *n* ordinary differential equations for the angular deviations of pendulums [31,32], where *n* is the number of pairs of pendulums in the system. In this case, the rotating pendulums correspond to nitrogenous bases, and the elastic thread to which these pendulums are attached correspond to the pentose-phosphate chains of the DNA molecule; the hydrogen bond of a pair of complementary nitrogenous bases corresponds to the elastic bond of a pair of pendulums.

We assume that a break occurs in the pair of bases if the potential energy of hydrogen bonds in this pair exceeds the critical value *E_AT_* for the *A-T* base pair and *E_GC_* for *G-C*; the bond is restored if its potential energy becomes less than the corresponding critical value [33,34].

The values of the mathematical parameters are taken from the works in [35,36]. The energy values for breaking hydrogen bonds in AT and GC pairs are as follows: *E_AT_* ≈ 5.1020 pN·nm and *E_GC_* ≈ 12.7064 pN·nm [37].

The amplitude of the torque effect *M*_0_ was designated as constant in time, and the spatial localization was selected from the range that was used in [28]. Solutions to the mathematical model were found numerically [38].

## 3. Results and Discussion

To reduce computational costs, a region of the *ATXN2* gene [39], containing 23 CAG repeats from 4601 to 6600 base pairs, is selected for calculations. The choice of such a section is justified by the fact that it contains the first exon of interest to us and, during the calculated period of time, the zone of disturbances caused by the applied torque does not reach the boundaries of the selected section, which allows us to adequately set boundary conditions. As the CAG tract expands, the right boundary of the calculated area increases accordingly.

At large values of the CAG repeat number (k), significant OS zones are formed in the promoter region at *M*_0_ = 8.28 pN∙nm. As the *M*_0_ value increases, additional OS zones may be formed in the CAG tract.

Calculations carried out for a wide range of values of the spatial localization of the torque and its value *M*_0_ show that the probability of the occurrence of additional OS zones of significant size increases with an increase in the number of CAG repeats, and the value inverse to the probability correlates with the average age of onset of the SCA2 disease [28]. In this work, the possibility of the formation of large additional OS zones in the CAG tract under torque will be called the stability of the CAG tract.

**Figure 1:**
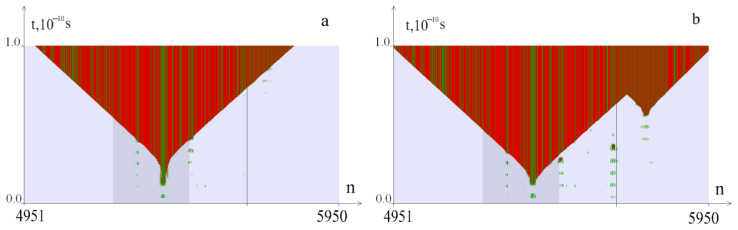
OS zones that arise during torsion action on the *ATXN2* gene region at k = 55. OSs in AT pairs are indicated in green, and in red—those in GC pairs. The promoter region is highlighted with a darker background. Note: (**a**) *M*_0_ = 8.28 pN∙nm—the OS zone originates in the promoter region; there is no additional OS zone; (**b**) *M*_0_ = 8.57 pN∙nm—an additional OS zone of significant size is formed in the CAG tract.

Figure 1 shows examples of the OS zones’ genesis at k = 55. OSs in AT pairs are indicated in green, and OSs in GC pairs are indicated in red. The promoter region is highlighted with a darker background; the line (5658th base pair) indicates the beginning of the CAG tract. In all figures, the numbers of nitrogen base pairs are shown horizontally, and time is shown vertically, which allows us to see the dynamics of the development of OS zones.

Figure 1a shows the OS zone formed at *M*_0_ = 8.28 pN∙nm, and Figure 1b—at *M*_0_ = 8.57 pN∙nm; it is clear that an additional OS zone of significant size is formed in the CAG tract.

To study the effect of CAA interruptions on the stability of the CAG tract, we will use parameter values selected from the range that was used in [28]. Calculations are carried out on the time interval [0, 10^−10^ s] for the number of CAG repeats k = 40, 45, 50, and 55 and the torque *M*_0_ = 8.57 pN∙nm. The boundaries of the spatial localization of the torque are counted from the beginning of the selected gene section; the left boundary is *i*_1_ = 633 (which coincides with the beginning of the promoter region), and the right boundary is *i*_1_ = 1225.

**Figure 2:**
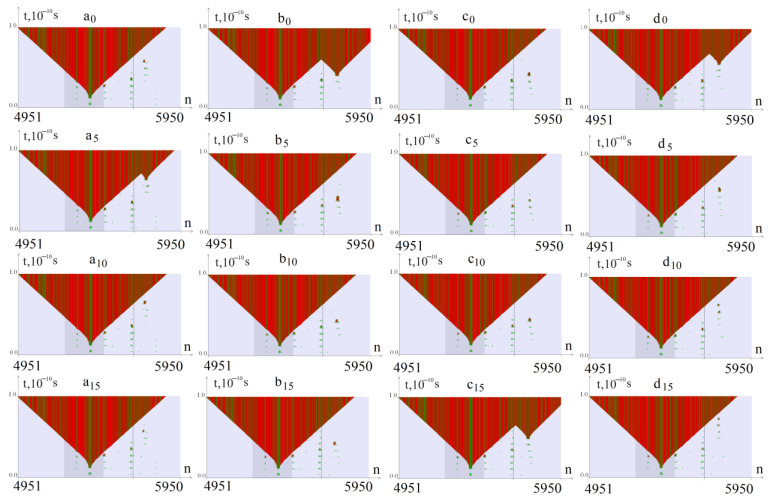
OS zones that arise during torsion action on a region of the *ATXN2* gene with the number of CAG repeats k = 40, 45, 50, and 55 (columns a, b, c, and d, respectively). The subscript of the letter indicates the number of the CAG trinucleotide replaced by the CAA trinucleotide (5, 10, 15, and 0—without a CAA interruption). OSs in AT pairs are indicated in green, and in GC pairs—in red. The promoter region is highlighted with a darker background. The value of the torsion moment *M*_0_ = 8.57 pN∙nm.

**Figure 3:**
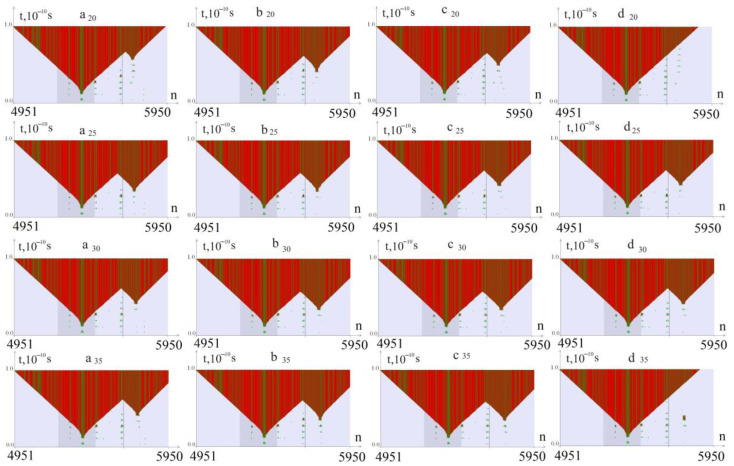
OS zones that arise during torsion action on a region of the *ATXN2* gene with the number of CAG repeats k = 40, 45, 50, and 55 (columns a, b, c, and d, respectively). The subscript of the letter indicates the number of the CAG trinucleotide replaced by the CAA trinucleotide (20, 25, 30, 35). OSs in AT pairs are indicated in green, and in GC pairs—in red. The promoter region is highlighted with a darker background. The value of the torsion moment *M*_0_ = 8.57 pN∙nm.

The calculation results of the CAA interruptions’ effect on the stability of the CAG tract are shown in Figure 2 and Figure 3. The columns marked with the letters a, b, c, and d show the OS zones that arise when torque is applied to the *ATXN2* gene region with the number of CAG repeats k = 40, 45, 50, and 55, respectively, the subscript of the letter indicates the number of the CAG trinucleotide replaced by the CAA trinucleotide (0—without CAA interruption).

From Figure 2, it can be seen that in the absence of CAA interruptions, additional OS zones appear in the CAG region at 45 and 55 repetitions. When replacing CAG repeats with CAA at positions 5, 10, and 15, a region-stabilizing effect occurs. Additional OS zones are not observed, except in the case of 50 CAG repeats and a CAA substitution at position 15.

From Figure 3, it can be seen that when replacing CAG repeats with CAA in positions 20, 25, 30, and 35, additional OS zones appear in almost all cases. A destabilizing effect occurs when CAA interruption occurs in the middle or right side of the CAG tract.

**Figure 4:**
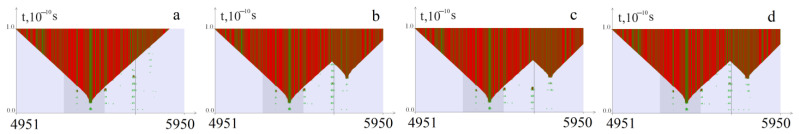
OS zones’ genesis under torque action in the *ATXN2* gene region with the number of CAG repeats k = 55 for several CAA interruptions: (**a**)—CAA trinucleotides replace 5 and 15 CAG trinucleotides; (**b**)—CAA trinucleotides replace 15 and 25 CAG trinucleotides; (**c**)—CAA trinucleotides replace 25 and 35 CAG trinucleotides; (**d**)—CAA trinucleotides replace 5, 15, 25, and 35 CAG trinucleotides. OS in AT pairs are indicated in green, and in GC pairs—in red. The promoter region is highlighted with a darker background. The value of the torque *M*_0_ = 8.57 pN∙nm.

Figure 4 shows the calculation results of several CAA interruptions’ effects on the CAG tract stability.

From Figure 4, it is clear that the presence of two CAA interruptions in the left part of the CAG tract causes a stabilization effect, and in the right part—a destabilization effect and the emergence of additional OS zones of significant size.

It is known that the CAG/CAA configuration influences the nature and time of disease onset caused by the expansion of trinucleotide repeats [8]. The considered examples show that under torque, CAA interruptions can significantly affect the stability of the CAG tract in the *ATXN2* gene. Moreover, the effect of CAA interruptions on the stability of the CAG tract depends on the location of the interruption: in the examples considered, an interruption on the left side leads, as a rule, to an increase in the stability of the CAG tract, and on the right—to its decrease (Figure 2 and Figure 3).

The presence of several CAA interruptions also affects the stability of the CAG tract (Figure 4). In the examples discussed, CAA interruptions on the left side increase the stability of the CAG tract. However, the presence of at least one CAA interruption on the right side of the CAG tract leads to a decrease in its stability.

## 4. Conclusions

This work shows that the torque effect on the *ATXN2* gene region containing the first exon, in addition to the formation of OSs in the promoter region, can lead to the formation of significant-size additional OS zones in the CAG tract. The probability of the occurrence of such zones increases with an increase in the number of CAG repeats.

A number of studies show that the mechanisms leading to the occurrence of diseases caused by the expansion of the CAG tract should be sought not only at the level of the physiological role of the *ATXN2* protein, but also at the DNA level [40,41,42]. These mechanisms are associated with non-canonical configurations (hairpins) that can form in the CAG tract. The tendency of hairpins to slide along corresponding threads is generally considered important in explaining the expansion of the CAG tract [17]. Research into the role of CAA interruptions has shown that, under certain conditions, they can stabilize hairpin dynamics by preventing the expansion of the CAG tract [40,43].

The number of CAA interruptions also affects the severity and nature of the disease [9]. Previously, authors Sobczak K. and Krzyzosiak W. J. asked the following question: what is the key factor influencing hairpin formation—the number or localization of CAA interruptions in the CAG tract [27]? We have established that the presence of CAA interruptions in the right part of the CAG tract most likely leads to the emergence of an additional OS zone. At the same time, if there are three CAA interruptions in the CAG tract, one or two interruptions will be located on the right side, which may contribute to the intensification of the disease. Thus, our data correlate with known results.

The calculations made it possible to establish that CAA interruptions affect the stability of the CAG tract, and this influence, depending on the location of the interruption, can either increase or decrease the stability of the CAG tract.

## Data Availability

The raw data supporting the conclusions of this article will be made available by the authors on request.
